# Supplementary material for: Patterns of health service utilisation of mothers experiencing mental health problems and intimate partner violence: Ten-year follow-up of an Australian prospective mother and child cohort
Source: PLoS One. 2022 Jun 15;17(6):e0269626. doi: 10.1371/journal.pone.0269626 (PMC9200341; doi:10.1371/journal.pone.0269626)
Supplement: S1 Table — (DOCX) [file pone.0269626.s001.docx]

**Supplementary Table 1. Comparison of the original cohort and women who completed ten-year follow-up**

|  | **Original** | **cohort** | **Participated in 10 year follow up** | | | | | |
| --- | --- | --- | --- | --- | --- | --- | --- | --- |
|  |  |  | Yes | | No | |  |  |
|  | n | % | n | % | n | % | Odds Ratio^1^ | 95%CI |
| Early pregnancy |  |  |  |  |  |  |  |  |
| Country of birth |  |  |  |  |  |  |  |  |
| Australia | 1115 | 74.4 | 744 | 78.3 | 371 | 67.6 | 1.0 | [ref] |
| Overseas – English speaking | 141 | 9.4 | 91 | 9.6 | 50 | 9.1 | 1.1 | [0.8,1.6] |
| Overseas – NESB speaking | 243 | 16.2 | 115 | 12.1 | 128 | 23.3 | 2.2 | [1.7,3.0] |
| Maternal employment |  |  |  |  |  |  |  |  |
| In paid employment | 1221 | 82.4 | 834 | 89.3 | 387 | 70.6 | 1.0 | [ref] |
| Not in paid employment | 261 | 17.6 | 100 | 10.7 | 161 | 29.4 | 3.5 | [2.6,4.6] |
| Maternal age |  |  |  |  |  |  |  |  |
| 18 – 24 years | 212 | 14.1 | 81 | 8.5 | 131 | 23.7 | 2.41 | [1.7,3.4] |
| 25 – 29 years | 436 | 29.0 | 261 | 27.4 | 175 | 31.6 | 1.0 | [ref] |
| 30 – 34 years | 579 | 38.5 | 419 | 44.0 | 160 | 28.9 | 0.57 | [0.4,0.7] |
| 35 + years | 278 | 18.5 | 191 | 20.1 | 87 | 15.7 | 0.68 | [0.5,0.9] |
| Maternal education level |  |  |  |  |  |  |  |  |
| Tertiary | 1081 | 72.1 | 732 | 77.2 | 349 | 63.3 | 1.0 | [ref] |
| Year 12 or less | 418 | 27.9 | 216 | 22.8 | 202 | 36.7 | 2.0 | [1.6,2.5] |
| Relationship status |  |  |  |  |  |  |  |  |
| Partnered | 1436 | 95.3 | 916 | 96.2 | 520 | 93.7 | 1.0 | [ref] |
| Not partnered | 71 | 4.7 | 36 | 3.8 | 35 | 6.3 | 1.7 | [1.1,2.8] |
| Family income (AUD) |  |  |  |  |  |  |  |  |
| >$100,000 per annum | 196 | 14.2 | 144 | 16.1 | 52 | 10.8 | 1.0 | [ref] |
| $60,001-$100,000 per annum | 435 | 31.6 | 321 | 35.8 | 114 | 23.8 | 1.0 | [0.7,1.4] |
| $40,001-$60,000 per annum | 519 | 37.7 | 332 | 37.0 | 187 | 39.0 | 1.6 | [1.1,2.2] |
| <$40,000 per annum | 227 | 16.5 | 100 | 11.1 | 127 | 26.5 | 3.5 | [2.3,5.3] |
| First year postpartum |  |  |  |  |  |  |  |  |
| Depressive symptoms (3, 6, 12mths) (EPDS) |  |  |  |  |  |  |  |  |
| <13 | 1215 | 83.5 | 813 | 85.4 | 402 | 79.9 | 1.0 | [ref] |
| ≥13 | 240 | 16.5 | 139 | 14.6 | 101 | 20.1 | 1.5 | [1.1,1.9] |
| Intimate partner violence (CAS) |  |  |  |  |  |  |  |  |
| No abuse | 1112 | 82.6 | 795 | 85.6 | 317 | 76.0 | 1.0 | [ref] |
| Abuse | 234 | 17.4 | 134 | 14.4 | 100 | 24.0 | 1.9 | [1.4,2.5] |
| **Total** | **1507** | **100.0** | **952** | **100.0** | **555** | **100.0** |  |  |

^1^ Odds Ratios reflect the odds of not participating in ten-year follow-up
